# Aspherical and Spherical InvA497-Functionalized Nanocarriers for Intracellular Delivery of Anti-Infective Agents

**DOI:** 10.1007/s11095-018-2521-3

**Published:** 2018-12-05

**Authors:** Arianna Castoldi, Martin Empting, Chiara De Rossi, Karsten Mayr, Petra Dersch, Rolf Hartmann, Rolf Müller, Sarah Gordon, Claus-Michael Lehr

**Affiliations:** 1 Department Drug Delivery, Helmholtz Institute for Pharmaceutical Research Saarland (HIPS), Helmholtz Center for Infection Research (HZI), 66123 Saarbrücken, Germany; 2 Department Drug Design and Optimization, HIPS, HZI, 66123 Saarbrücken, Germany; 3 Department Microbial Natural Products, HIPS, HZI, 66123 Saarbrücken, Germany; 4 Department Molecular Infection Biology, HZI, 38124 Braunschweig, Germany; 5 School of Pharmacy and Biomolecular Sciences, Liverpool John Moores University, Liverpool, L3 3AF UK; 6 Department of Pharmacy, Saarland University, 66123 Saarbrücken, Germany

**Keywords:** aspherical nanoparticles, AOT-gentamicin, bacteriomimetic nanocarriers, intracellular infection, invasin

## Abstract

**Purpose:**

The objective of this work was to evaluate the potential of polymeric spherical and aspherical invasive nanocarriers, loaded with antibiotic, to access and treat intracellular bacterial infections.

**Methods:**

Aspherical nanocarriers were prepared by stretching of spherical precursors, and both aspherical and spherical nanocarriers were surface-functionalized with the invasive protein InvA497. The relative uptake of nanocarriers into HEp-2 epithelial cells was then assessed. Nanocarriers were subsequently loaded with a preparation of the non-permeable antibiotic gentamicin, and tested for their ability to treat HEp-2 cells infected with the enteroinvasive bacterium *Shigella flexneri*.

**Results:**

InvA497-functionalized nanocarriers of both spherical and aspherical shape showed a significantly improved rate and extent of uptake into HEp-2 cells in comparison to non-functionalized nanocarriers. Functionalized and antibiotic-loaded nanocarriers demonstrated a dose dependent killing of intracellular *S. flexneri*. A slight but significant enhancement of intracellular bacterial killing was also observed with aspherical as compared to spherical functionalized nanocarriers at the highest tested concentration.

**Conclusions:**

InvA497-functionalized, polymer-based nanocarriers were able to efficiently deliver a non-permeable antibiotic across host cell membranes to affect killing of intracellular bacteria. Functionalized nanocarriers with an aspherical shape showed an interesting future potential for intracellular infection therapy.

**Electronic supplementary material:**

The online version of this article (10.1007/s11095-018-2521-3) contains supplementary material, which is available to authorized users.

## Introduction

While delivery of anti-infective drugs using nanocarriers is an attractive option for the treatment of infections, several factors may act to limit the efficacy of this strategy. The specific location of bacteria constitutes one such obstacle – many common enteropathogenic bacteria such as *Salmonella, Shigella* and *Yersinia* spp. are able to invade and replicate inside host cells, where, due to the common poor membrane permeability of many anti-infective agents, they prove difficult to reach (1,2). Incorporation of drug candidates into particulate nanocarriers functionalized with invasive moieties to enhance cellular uptake is a potential way to overcome this delivery problem. In this respect, the use of bacterial proteins which naturally mediate the invasion of bacteria into mammalian cells has been reported as a promising means of enhancing the permeation of carrier systems, and potentially increasing the intracellular efficacy of their drug loads (3–6).

A particularly interesting candidate in this category is invasin, a well-characterized outer membrane invasion protein expressed on the surface of *Yersinia pseudotuberculosis* and *Yersinia enterocolitica*, which mediates an efficient entry of the bacteria into eukaryotic cells through interaction with β_1_ integrin receptors (7,8). The last 497 amino acids of the *C-*terminal region of invasin have been found to be particularly important for receptor binding and intracellular uptake (8). Dersch *et al*. were able to produce and purify such a *C-*terminal, cell-invasive fragment of invasin, referred to as InvA497 (8); gentamicin-loaded liposomes surface functionalized with InvA497 were further demonstrated to be able to reach and kill intracellular bacteria located in various epithelial sub-cellular compartments (3,9).

The potential of invasin and the invasin fragment InvA497 is therefore clear, however the full capacity of so-called bacteriomimetic systems, functionalized with such cell invasion-promoting molecules, still remains to be fully explored. In the first instance, while surface functionalization of comparatively more robust, polymer-based carrier systems with invasin has been shown to improve their cellular uptake (5,6), drug loading of such systems and investigation of their ability to affect intracellular delivery of actives has not been investigated in-depth. In addition to probing the ability of invasin to mediate effective intracellular delivery of drug loads from carrier systems composed of a variety of materials, there is also a need to investigate the role of other factors, such as the shape of these carrier systems, on delivery efficacy.

There is clear evidence of the importance of the shape of colloidal structures in biological interactions, including the shape variation of bacteria themselves (10,11); however, due to the fact that most nanoparticles employed for drug delivery are inherently spherical (as a result of the nature of utilized preparation procedures), the role of their shape in mediating effective drug delivery has largely been unexplored to date (12,13). Studies have shown the possibility of using particles with an aspherical shape to alter circulation time and biodistribution (14,15) as well as cellular internalization and trafficking (16–20), subsequently influencing the interaction of the particle with its target. With respect to cell uptake, particle shape effects have been mainly studied in phagocytic cells such as macrophages, as well as non-phagocytic epithelial cells - and in most cases, the uptake of elongated particles was significantly inhibited in comparison to spherical controls (19,21,22). In contrast, initial studies looking at the influence of shape on uptake by non-phagocytic epithelial cells have shown that aspherical nanoparticles surface-functionalized with biotin had an enhanced uptake into human enterocytes (23), and that trastuzumab-coated nanorods had a higher uptake in breast cancer cell lines than spherical or disk-shaped nanoparticles (24). The efficacy of loaded aspherical systems has also been investigated, with varying results. Kolhar *et al*. have shown a positive effect of elongated particles with surface-adsorbed antibody or protein on targeting the endothelium, for delivery of chemotherapeutics (25). Hinde *et al*. have also shown the advantages of nanoparticles with high aspect ratios, such as ‘worms’ and rods, for delivery of doxorubicin into the nuclei of epithelial breast cancer cells, demonstrating the impact of various nanocarrier shapes on anticancer formulations (26). When investigated for their potential as vaccine delivery systems however, elongated nanoparticles were shown to be inferior to spherical nanoparticles with respect to dendritic cell activation (18). Therefore, although shape has a clear impact on delivery system behavior, the nature of this impact may vary. As such, the effect of aspherical shaped delivery systems must be carefully evaluated in relation to factors including the surface composition of the particulate system, the specific target cell, the drug delivered and the nature of the specific delivery system application. As mentioned above, the uptake of aspherical systems has been investigated in a variety of cell types, however to date the efficacy of drug-loaded aspherical nanoparticles has mainly been studied for delivery of chemotherapeutics; intracellular delivery of other drug classes, such as antibiotics, using aspherical nanoparticles has not yet been investigated to the best of the authors´ knowledge.

Therefore, the objectives of this study were to investigate the cellular uptake and efficacy of drug-loaded, polymeric bacteriomimetic systems against intracellular bacteria, and to determine the influence of shape on the physico-chemical characteristics of these systems. For this purpose, InvA497-functionalized polymeric nanoparticles with spherical and aspherical morphologies were prepared. Bacteriomimetic systems were shown to have a greater uptake into cells of the HEp-2 human epithelial cell line than non-functionalized nanoparticles, regardless of shape. HEp-2 epithelial cells, which could be reproducibly infected with intracellular *Shigella flexneri*, were then used as a model for efficacy testing of bacteriomimetic systems loaded with a lipophilic preparation of the antibiotic gentamicin. InvA497-functionalized and drug-loaded systems were able to penetrate into infected cells and kill intracellular *S. flexneri*, while negligible cell uptake and bacterial killing was demonstrated by the non-functionalized nanoparticles. Moreover, a slight but significant improvement in bacterial killing was found following treatment with high dose aspherical bacteriomimetic systems, in comparison to spherical. The current work therefore represents the first study into the impact of modifying the surface of drug-loaded, polymeric nanoparticles through the use of the bacteria-derived invasion molecule InvA497, in combination with investigation of carrier system shape effects.

## Materials and Methods

### Materials

For the preparation and storage of spherical and aspherical nanoparticles, poly(lactic-co-glycolic-acid) (PLGA, Resomer RG 503 H, lactic/glycolic acid 50/50 wt/wt; molecular weight 40.3 kDa; inherent viscosity 0.41 dl/g; Evonik Industries AG, Darmstadt, Germany), polyvinyl alcohol (PVA; Mowiol® 4–88, Kuraray Specialties Europe GmbH, Frankfurt, Germany), trehalose (Sigma-Aldrich, Steinheim, Germany) and glycerol (Sigma-Aldrich, Steinheim, Germany) were used. As a coupling agent, 4-(4,6-Dimethoxy-1,3,5-triazin-2-yl)-4-methylmorpholinium chloride (DMTMM, Sigma Aldrich, Steinheim, Germany) was employed. For *in vitro* cell experiments HEp-2 cells (ATCC, Manassas, USA), Roswell Park Memorial Institute (RPMI) 1640 medium (Gibco, Carlsbad, USA), fetal calf serum (FCS, Lonza, Cologne, Germany), rhodamine-labeled *Ricinus communis* Agglutinin I (Vector Laboratories, Inc., Burlingame, CA, USA), paraformaldehyde (Electron Microscopy Sciences, Hatfield, USA) and 4′,6-diamidino-2-phenylindole (DAPI, stock 1 mg/ml; LifeTechnologies™, Darmstadt, Germany) were purchased. For quantification of gentamicin, o-phthaldialdehyde reagent (OPA, Sigma Aldrich, Steinheim, Germany) was used. As an intracellular bacterium for infection studies, *S. flexneri* (clinical strain M90 T) was kindly supplied by the Department of Molecular Infection Biology, HZI, Braunschweig, Germany. For efficacy studies gentamicin (Sigma-Aldrich, Steinheim, Germany) and Triton X-100 (Sigma Aldrich, Steinheim, Germany) were purchased. Distilled de-ionized water with conductivity of less than 18.2 MΩ/cm at 25°C and organic solvents of HPLC grade were used for all experiments.

### Preparation of Spherical Nanoparticles

Spherical PLGA nanoparticles were prepared using a single emulsion method (27), employing a mixture of PLGA and fluoresceinamine-PLGA (FA-PLGA) prepared according to Weiss *et al*. (28) at a 0.25:0.75 weight ratio. Briefly, the polymer mixture was dissolved in 2 ml ethyl acetate (20 mg/ml) and sonicated with 4 ml of a 2% (*w*/*v*) PVA solution at 12 W for 30 s (Digital sonifier 450, Branson Ultrasonic Corporation, Danbury, USA). After adding 15 ml of water, the formed emulsion was left to stir overnight to allow for solvent evaporation. Excess PVA was then removed from the nanoparticle dispersion by centrifugation (10,000 *g* for 12 min at 12°C). Nanoparticle suspensions were then stored at 4°C for maximum 1 week prior to further use, or freeze dried (Alpha 2–4 LSC, Christ, Osterode am Harz, Germany) with 0.31 mg/ml of trehalose as cryoprotectant and stored at room temperature.

### Aspherical Nanoparticle Preparation

Aspherical nanoparticles were prepared according to a previously described film stretching method (29,30). Briefly, 0.1% (*w*/*v*) of spherical nanoparticles was mixed with a solution of 10% PVA and 2% (*v*/v) glycerol. The resulting dispersion was dried overnight in a mold in order to create a flat, dry film. Sections of the film were then immobilized in an in-house fabricated stretching machine, which was immersed in mineral oil at 54°C (above the PLGA glass transition temperature). The films were then stretched longitudinally to twice their original length. The stretched films were allowed to cool down, and after removing the excess oil by washing with isopropanol, were dissolved in water. PVA was removed and the stretched, aspherical nanoparticles purified using multiple cycles of high speed centrifugation (16,000 *g* for 20 min at 12°C) followed by centrifugation (5–6 cycles of 10 min at 1179 *g*, 4°C) with Centrisart® ultrafiltration tubes (300,000 molecular weight cut off, Sartorius, Göttingen, Germany). Aspherical nanoparticles were stored at 4°C until further use.

### Characterization of Nanoparticles

The morphology of spherical and aspherical nanoparticles was characterized using scanning electron microscopy (SEM, Zeiss EVO HD 15, Carl Zeiss AG, Oberkochen, Germany) employing an accelerating voltage of 5 kV. Prior to analysis samples were diluted and dried overnight, before being sputter coated (Quorum Q150R ES, Quorum Technologies Ltd., Laughton, United Kingdom) with gold. Physical characterization of spherical nanoparticle dispersions was performed by dynamic light scattering (DLS) using a Zetasizer Nano (Malvern Instruments Ltd., Worcestershire, United Kingdom). For aspherical nanoparticles, the common shape descriptors of major and minor axis length and aspect ratio (AR) were measured from SEM images using ImageJ software (Fiji).

### Surface Functionalization

Nanoparticles were functionalized with InvA497, a fragment of the *Y. pseudotuberculosis* invasin protein, consisting of 497 amino acids of the parent protein *C*-terminus (3,31).

The InvA497 fragment was purified as described previously (31). In the case of spherical nanoparticles, a volume of 1 ml of spherical PLGA nanoparticles was diluted with 0.9 ml water, and incubated for 2 h with a 5 mg/ml solution of the carboxyl group activating agent DMTMM at room temperature. Afterwards the dispersion was again diluted in water and InvA497 was added to a final concentration of 320 μg/ml, followed by overnight stirring in an ice bath. To remove excess DMTMM and unbound InvA497, nanoparticles were then centrifuged three times in Centrisart® tubes as mentioned above, at 1605 *g* and 4°C, for 10 min each cycle.

In order to produce InvA497-functionalized aspherical nanoparticles, spherical PLGA nanoparticles were first incubated for 2 h with DMTMM as described above. Particles were then immobilized in PVA-glycerol films, and stretched using the stretching apparatus as described above. A 2.5 ml volume of the resulting aspherical nanoparticle dispersion was then stirred overnight in an ice bath with InvA497 (concentration 320 μg/ml). To remove unbound InvA497, the dispersion was then centrifuged in Centrisart® tubes at 1605 *g* and 4°C, for three cycles of 10 min each.

### Quantification of Functionalized InvA497

The amount of InvA497 coupled to spherical and aspherical nanoparticles was quantified using a bicinchoninic acid (BCA) kit (QuantiPro™; Sigma-Aldrich, Steinheim, Germany), in accordance with the manufacturer’s instructions and as previously described (3,32).

### Nanoparticle Counting

The number of spherical and aspherical InvA497-functionalized nanoparticles within defined samples was counted using nanoparticle tracking analysis (NTA, NanoSight LM 10, Malvern Instruments Ltd., Worcestershire, United Kingdom). After appropriate dilution, the concentration of nanoparticles within a sample was first calculated by the NTA software, and then converted into a number of nanoparticles in the total dispersion. The extrapolated number of nanoparticles was then combined with the quantified amount of InvA497 present on nanoparticle surfaces, and used to estimate the number of InvA497 molecules per nanoparticle as well as the protein density on nanoparticle surfaces.

### Generation of 3D Model

A 3D model of InvA497-decorated spherical and aspherical nanoparticles was generated via a self-written script for PovRay 3.7. To this end, X-ray coordinates of InvA497 (PDB ID: 1CWV) (33) were exported and scaled to the PovRay format using YASARA structure (YASARA Biosciences) (34). After modeling of the nanoparticulate shapes, the calculated numbers of 198 and 235 InvA497 molecules were randomly distributed on the aspherical and spherical objects, respectively. The picture was rendered with subsurface light scattering turned on.

### Cell Culture

Cells of the human larynx carcinoma-derived HEp-2 cell line were employed for both uptake and efficacy studies. HEp-2 cells (passage number 10–18) were cultured in 75 cm^2^ flasks using RPMI 1640 medium, supplemented with 10% FCS. Cells were incubated at 37°C and 5% CO_2_ and medium was changed every two to three days. Cells were split upon 80% confluency.

### Uptake Studies

HEp-2 cells were seeded onto 24-well cell culture plates the day before conduction of uptake studies. For the studies themselves, cells were incubated with non-loaded spherical and aspherical nanoparticles, with or without surface coupled InvA497 (455 μg/ml of PLGA and, where appropriate, 60 μg/ml of InvA497 per sample) for time points ranging between 1 and 5 h, at 37°C and 5% CO_2_.

For confocal imaging of nanoparticle-treated cell samples, the supernatant was first removed from culture plate wells and cells were washed twice in order to remove non-internalized nanoparticles. Cell membranes were then stained with 20 μg/ml rhodamine-labeled *Ricinus communis* Agglutinin I. After fixation with 3% paraformaldehyde in phosphate buffered saline (PBS), cell nuclei were stained with DAPI (stock 1 mg/ml) diluted 1:50000 in PBS. Cells were imaged using confocal laser scanning microscopy (CLSM, Leica TCS SP 8; Leica, Mannheim, Germany). Analysis of gained images was performed using LAS X software (Leica Application Suite X; Leica, Mannheim, Germany).

For fluorescence-activated cell sorting (FACS) analysis, after removing cell supernatants and washing to remove extracellular nanoparticles, cells were detached from 24-well culture plates by incubating with 100 μl of 0.05% trypsin-EDTA (1x, Gibco, Carlsbad, USA) for 10 min at 37°C. A 900 μl volume of 2% FCS in PBS was then added, and cell samples were centrifuged at 257 *g* for 5 min at 4°C. Cell pellets were then resuspended in a 600 μl volume of 2% FCS in PBS, and stored at 4°C until FACS analysis. Flow cytometry was performed using a BD LSRFortessaTM (BD Biosciences, Heidelberg, Germany) using BD FACSDiva™ Software v8.0.1. Forward scatter (FSC) and side scatter (SSC) were collected for live cells within samples, using an untreated negative control for reference. From living cell populations, green fluorescence data (ex: 488 nm, filter: 530/30) were collected on a minimum of 10,000 events (cells) per sample.

To determine the energy dependence of nanoparticle uptake, the same uptake study procedure and FACS analysis was applied, after also incubating the nanoparticles with HEp-2 cells at 4°C.

### Preparation of AOT-Gentamicin Loaded Nanoparticles

Spherical and aspherical nanoparticles were loaded with a lipophilized preparation of gentamicin (gentamicin bis(2-ethylhexyl) sulfosuccinate sodium salt, or AOT-gentamicin), prepared according to Imbuluzqueta *et al*. (35,36). Spherical AOT-gentamicin nanoparticles (SphG) were first prepared as described above, incorporating 3 mg of the ionic AOT-gentamicin preparation into the PLGA solution prior to emulsion formation. The unentrapped AOT-gentamicin was removed using centrifugation and precipitation (10,000 *g* for 12 min at 12°C). Aspherical AOT-gentamicin (AsphG) nanoparticles were prepared by stretching of SphG nanoparticles, as described above. Where required, InvA497 was then coupled on the surface in order to produce functionalized spherical and aspherical nanoparticles loaded with AOT-gentamicin (respectively SphIG and AsphIG), also as described above.

The amount of AOT-gentamicin encapsulated in nanoparticles was quantified as described by Imbuluzqueta *et al*. (36) using a fluorometric method based on the use of OPA. The amount of encapsulated AOT-gentamicin within samples together with the initial amount of drug used during nanoparticle preparation was used to calculate nanoparticle encapsulation efficiency (EE%). After determining the dry mass of nanoparticle samples, the loading capacity (LC%) was calculated as the amount of encapsulated drug related to the total sample weight.

### *In Vitro* Drug Release Testing

Multiple samples of nanoparticle formulations containing 69.36 μg/ml of AOT-gentamicin were centrifuged and resuspended in 7 ml of PBS pH 7.4 in order to achieve sink conditions. Formulation samples were incubated at 37°C under stirring for 48 h; at various time points, 2 samples of each nanoparticle formulation were taken and centrifuged (1680 *g*, 10 min) in order to sediment the drug-containing nanoparticles. From the produced supernatant, the amount of released AOT-gentamicin was measured using the aforementioned fluorometric method (36). Release testing was conducted in triplicate, independent experiments.

### Efficacy Studies

The ability of drug-loaded nanoparticle systems to kill intracellular bacteria was tested in *S. flexneri*-infected HEp-2 epithelial cells. After seeding and culturing HEp-2 cells in a 24-well culture plate for 24 h, the cells were infected with *S. flexneri* dispersed in RPMI 1640 medium as described in the Supplementary Material (MOI of 25:1, CFU approximately 1.8 × 10 ^3^). Cells were then washed with PBS and incubated for 2 h with RPMI medium containing 50 μg/ml of gentamicin for extracellular bacteria killing. After killing of extracellular bacteria, infected HEp-2 cells were incubated with the InvA497-functionalized or non-functionalized, spherical and aspherical AOT-gentamicin loaded nanoparticles (120 μg/ml of AOT-gentamicin, 49 μg/ml of InvA497 and 3.7 mg/ml of PLGA/FA-PLGA where appropriate – standardized by employing drug-free functionalized or drug-free non-functionalized nanoparticles of corresponding shape where necessary) or with AOT-gentamicin alone, for 3 h at 37°C and 5% CO_2_. HEp-2 cells were then lysed using 0.01% Triton X-100 and the cell lysate was plated in sterile agar plates in serial dilutions. Plated lysates were incubated overnight at 37°C; after counting *S. flexneri* bacterial colonies and multiplication by relevant dilution factors, the final number of colony forming units (CFU) calculated from each cell lysate was expressed as the percentage of remaining intracellular bacteria (relative to the CFU of bacteria used for initial infection – the inoculum). Values for each formulation treatment group were then normalized to the percentage of remaining intracellular bacteria in untreated cell samples, and expressed as a percentage of bacterial killing.

### Statistical Analysis

Where appropriate, data are expressed as mean ± standard error of mean (SE). Also where appropriate, data was analyzed using SigmaPlot Version 11 (Systat Software Inc., San Jose, CA, USA) for statistical significance. Comparisons between groups were performed using Student’s t test (two-sided) or one-way ANOVA with post-hoc Bonferroni adjustment for experiments with more than two subgroups. Results were considered statistically significant at *p* values <0.05.

## Results

### Nanoparticle Preparation and Characterization

**Fig. 1:**
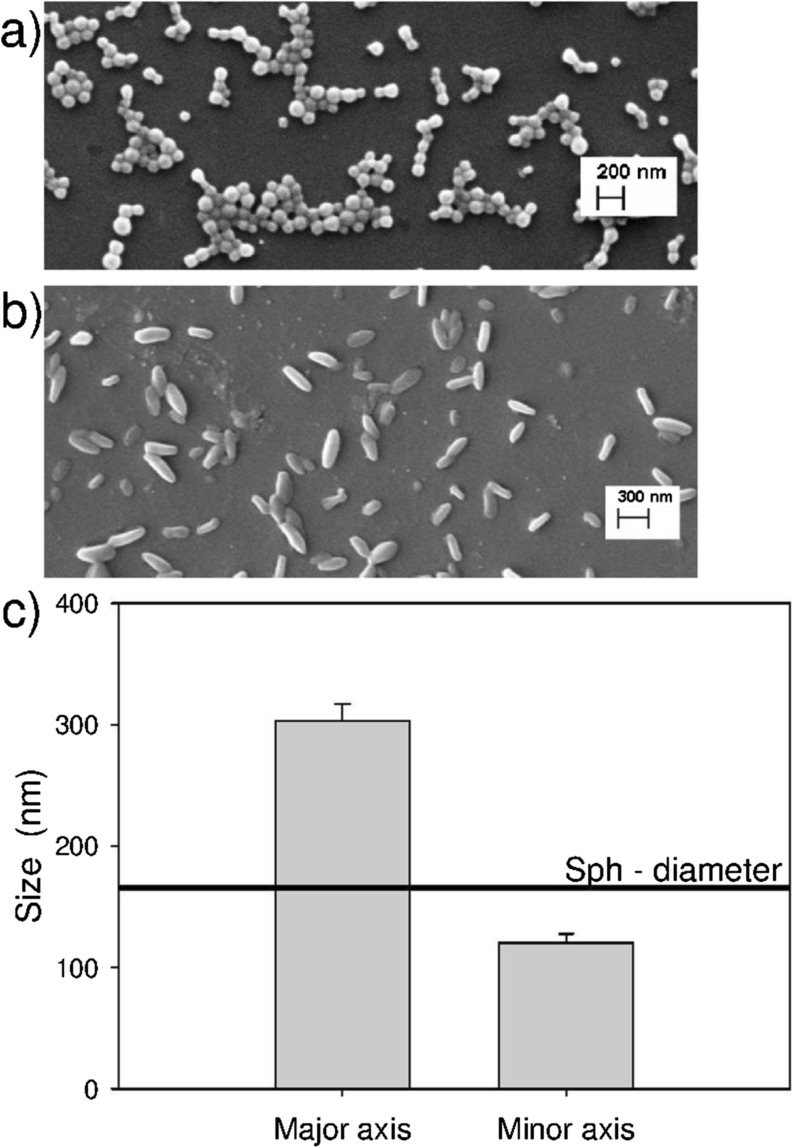
Characterization of spherical and aspherical nanoparticles. SEM images of spherical (**a**) and aspherical (**b**) nanoparticles; (**c**) size of the aspherical nanoparticles (‘Asph’) with respect to their major and minor axis, with the mean diameter of spherical nanoparticles (‘Sph’ – 164.8 ± 8.3 nm) given as reference. Results represent the mean ± SE (*n* = 200).

Spherical (Sph) nanoparticles produced using a mixture of PLGA and FA-PLGA were stretched, employing a film stretching method established by Champion *et al*. (29), in order to produce aspherical (Asph) nanoparticles. A change in the shape of stretched nanoparticles was confirmed by SEM imaging (Fig. 1a and b). Nanoparticle dimensions were then determined by SEM image analysis for Asph nanoparticles, giving values of approximately 300 nm and 110 nm for major and minor particle axes respectively (Fig. 1c), and an AR of 2.6. Sph nanoparticles were by comparison approximately 164.8 nm in diameter, as determined by DLS (Fig. 1c).

**Fig. 2:**
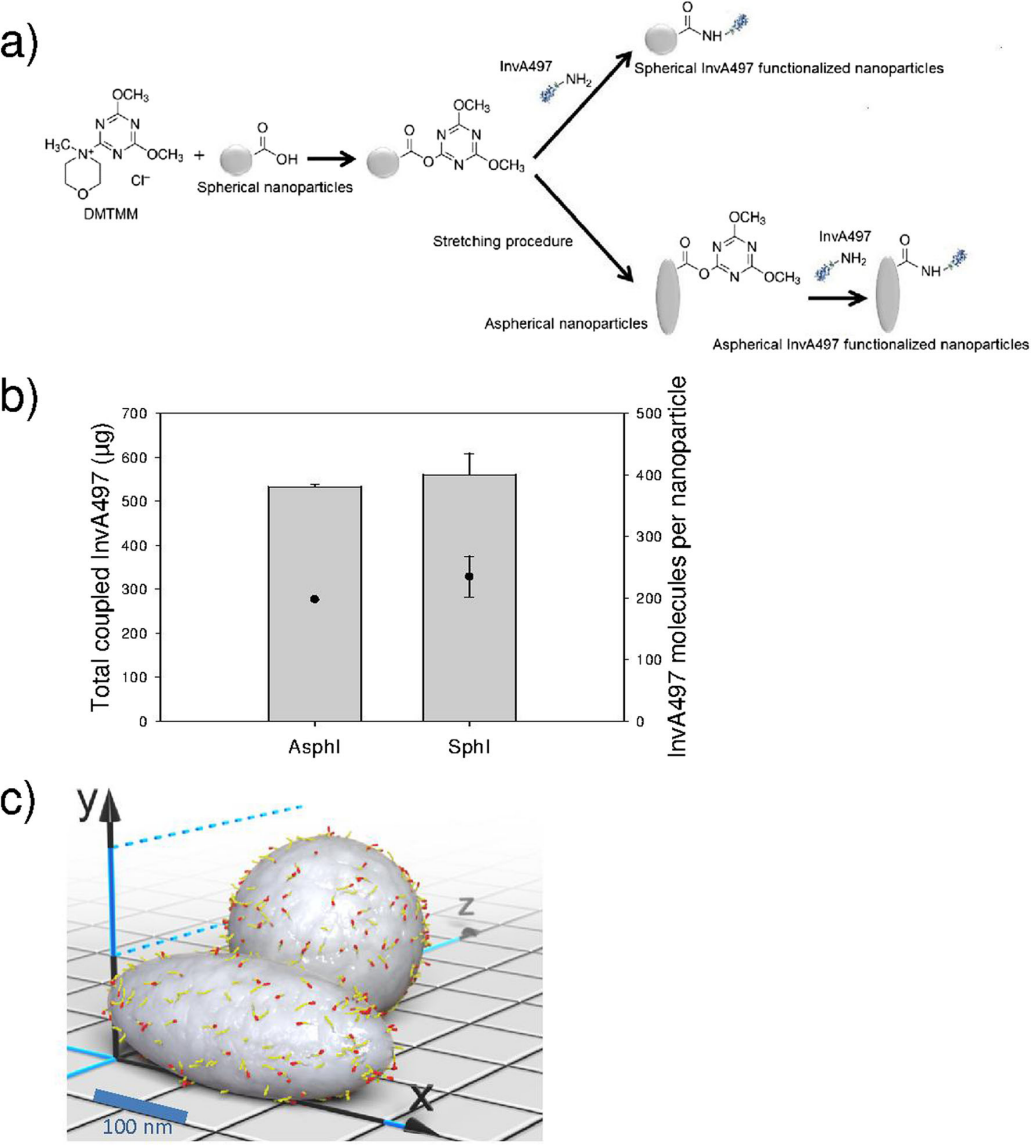
Characterization of InvA497 functionalized nanoparticles. (**a**) Schematic of functionalization procedure of the nanoparticle surface with InvA497: carboxyl groups of spherical nanoparticles were first activated with 4-(4,6-Dimethoxy-1,3,5-triazin-2-yl)-4-methylmorpholinium chloride (DMTMM) and then InvA497 was coupled on the surface. For aspherical nanoparticles, surface coupling of InvA497 was performed after activation of spherical nanoparticle carboxylic acid groups and particle stretching; b) quantification of the total amount of coupled InvA497 (grey bars) and estimation of the number of InvA497 molecules/nanoparticle (black circles), for aspherical (AsphI) and spherical (SphI) nanoparticles; c) 3D model of AsphI and SphI. The scale of the particles and the number of surface InvA497 molecules corresponds to the experimentally determined values. InvA497 molecules are shown in yellow, while their C-terminal integrin-binding regions are highlighted in red. Particle surfaces are colored grey. Where appropriate results represent the mean ± SE (*n* = 3).

Functionalization of both aspherical and spherical nanoparticles with InvA497, a *C-*terminal fragment of the bacterial invasion protein invasin, was then achieved by reacting amine functionalities of InvA497 with free PLGA carboxyl groups on nanoparticle surfaces (Fig. 2a). A continued aspherical and spherical particle shape following the coupling procedure was confirmed using SEM imaging, and the size of both functionalized particle formulations was found to be comparable to non-functionalized nanoparticles (Fig. S1). Coupling conditions were optimized so that both the total amount and the number of coupled InvA497 molecules/nanoparticle were comparable for aspherical and spherical nanoparticles (formulations AsphI and SphI respectively, Fig. 2b). Approximately 200 molecules of InvA497 were determined to be present on the surface of each AsphI or SphI nanoparticle. Using published X-ray coordinates of InvA497 (PDB ID: 1CWV) (33) as well as the experimentally determined scales and proportions of both particle types (vide supra), illustrative 3D models of the corresponding surface-modified carrier systems were generated (Fig. 2c).

Assuming that particle surface areas can be calculated by applying the generic formulas of the corresponding geometric shapes (Sph: sphere and Asph: spheroid) and that each InvA497 molecule occupies a circular area dictated by its gyration radius, the degree of particle surface coverage was estimated. According to these assumptions, approximately 39% of the surface of optimized AsphI and SphI nanoparticles is occupied by the InvA497 protein (for calculation see Supplementary Material).

### Nanoparticle Uptake

**Fig. 3:**
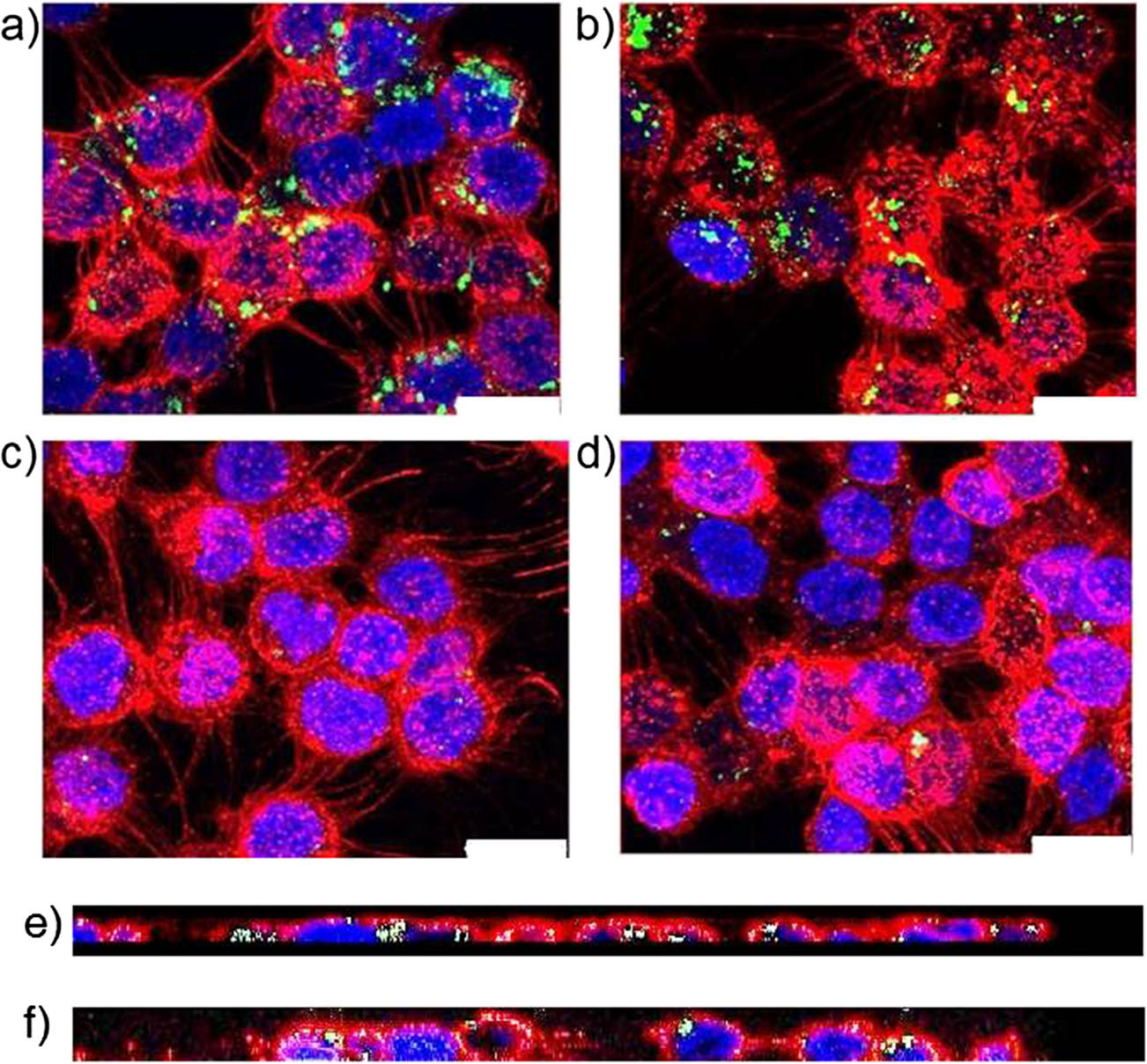
Confocal microscopy images of the cellular uptake of nanoparticles. Uptake of aspherical (‘AsphI’, **a**) and spherical (‘SphI’, **b**) nanoparticles functionalized with InvA497, as well as non-functionalized aspherical (‘Asph’, **c**) and spherical (‘Sph’, **d**) control nanoparticles into HEp-2 cells is shown after 5 h of incubation at 37°C. Cross sections for a (**e**) and b (**f**) are also shown, demonstrating the internalization of the nanoparticles inside the HEp-2 cells. Red: HEp-2 cell membranes, green: nanoparticles, blue: HEp-2 cell nuclei. Scale bar: 20 μm.

**Fig. 4:**
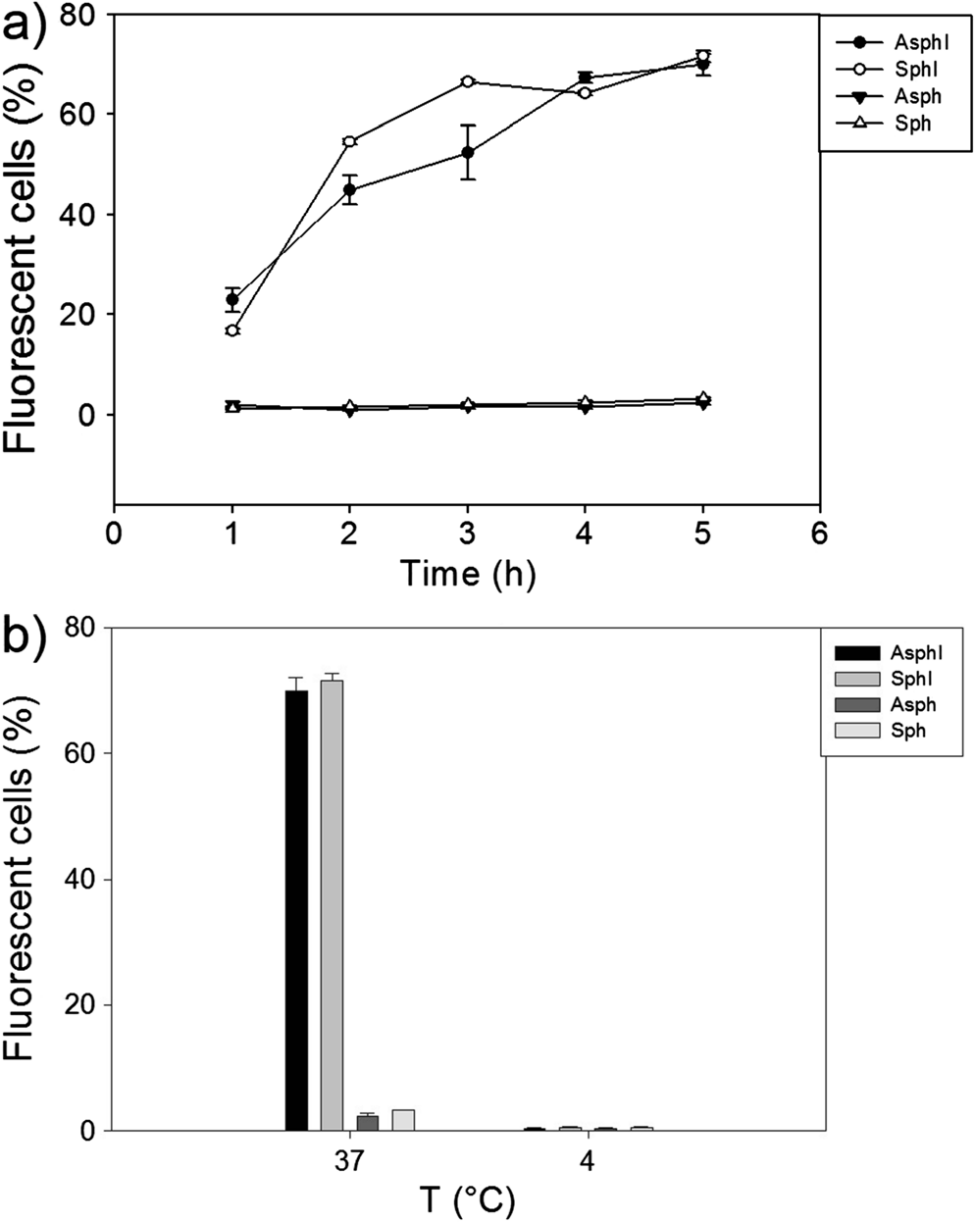
Quantification of intracellular uptake of nanoparticles. a) Flow cytometry was employed to quantify nanoparticle uptake into HEp-2 cells and to analyze the uptake kinetics of aspherical InvA497-functionalized (AsphI) and spherical InvA497-functionalized (SphI) nanoparticles, as well as non-functionalized aspherical (Asph) and spherical (Sph) particles as controls. Uptake of AsphI and SphI was significantly greater than Asph and Sph at all investigated time points (*p* < 0.001). b) Uptake of AsphI, SphI, Asph and Sph nanoparticles after 5 h of incubation with HEp-2 cells at 37°C and 4°C was also investigated, in order to assess the energy dependence of particle uptake. Results represent the mean ± SE (*n* = 3).

Prior to carrying out uptake studies, the cytotoxicity of functionalized and non-functionalized aspherical and spherical nanoparticles was preliminarily tested in a HEp-2 epithelial cell model (Fig. S2). Functionalized and non-functionalized aspherical and spherical nanoparticles were incubated with HEp-2 cells and the uptake was assessed by both CLSM (Fig. 3) and FACS analysis (Fig. 4).

Confocal images clearly demonstrated an increased uptake of AsphI and SphI (Fig. 3a and b respectively) in comparison to Asph and Sph formulations (Fig. 3c and d) at the endpoint of uptake studies, as well as the localization of nanoparticles inside the cells (Fig. 3e and f).

The kinetics of particle uptake were then quantified using FACS in order to further investigate the influence of particle functionalization and shape on cellular internalization (Fig. 4a). Within the first 3 h, uptake of InvA497-functionalized nanoparticles was found to be significantly greater than the non-functionalized carriers independent of particle shape. SphI demonstrated a slightly faster uptake than AsphI in the early stages of the study; by *t* = 4 h however, the uptake ratio had leveled out at approximately the same value of 66% and, hence, became independent of particle geometry.

To investigate if particle uptake occurred as a result of energy dependent endocytosis or rather by membrane association, the uptake of various particle formulations into HEp-2 cells at 37°C and 4°C was compared (Fig. 4b). A significant reduction in the percentage of fluorescent cells was observed for both AsphI and SphI at 4°C in comparison to 37°C.

### Nanoparticle Loading with AOT-Gentamicin

**Fig. 5:**
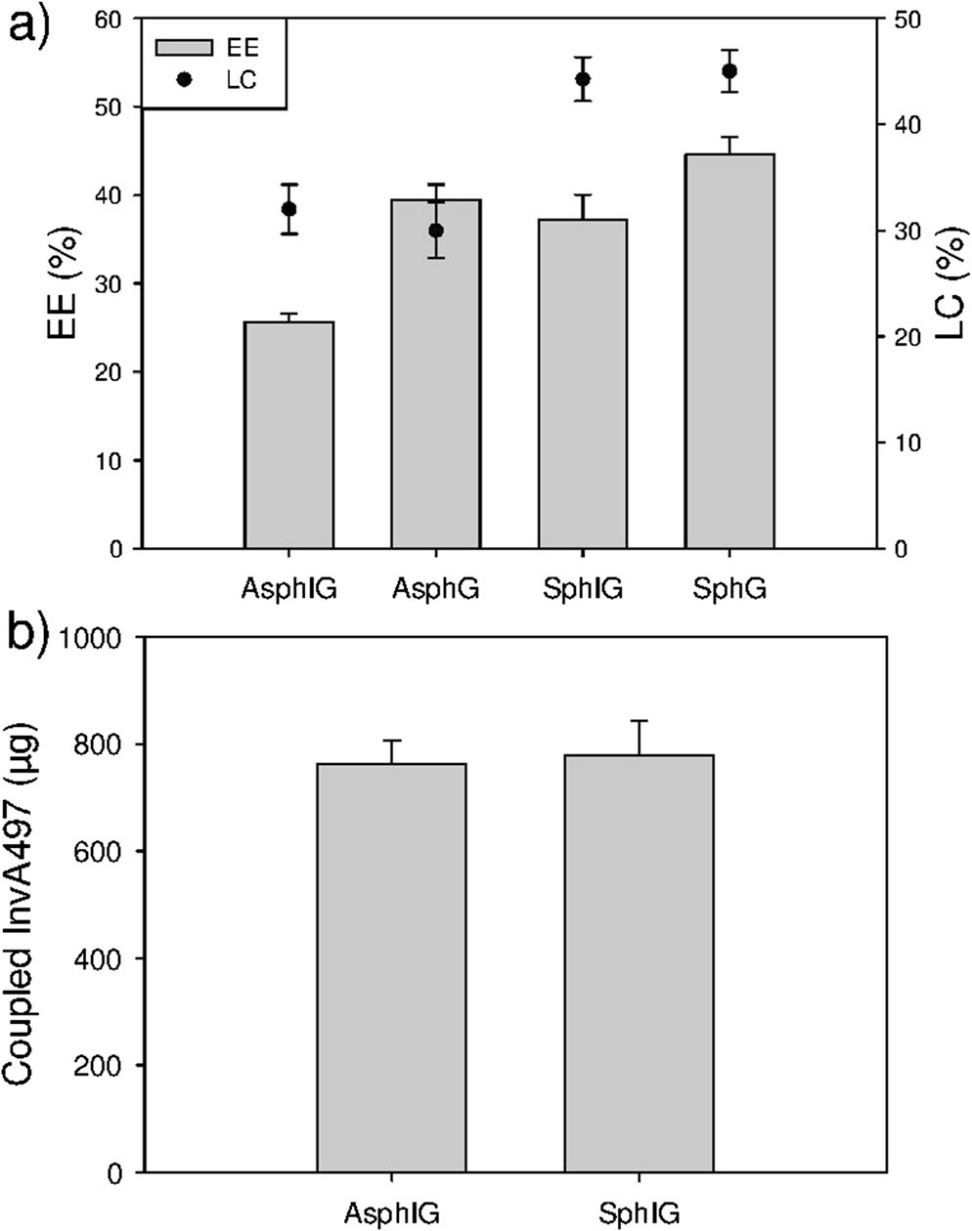
Characterization of drug-loaded nanoparticles. (**a**) The encapsulation efficiency (EE%, bars) and loading capacity (LC%, circles) of aspherical InvA497-functionalized, AOT-gentamicin loaded nanoparticles (AsphIG) and spherical InvA497-functionalized, AOT-gentamicin nanoparticles (SphIG), as well as aspherical and spherical drug-loaded nanoparticles which were not functionalized with InvA497 (AsphG and SphG respectively) is shown. (b) The amount of InvA497 coupled onto the surface of AsphIG and SphIG formulations was shown to be comparable. Results represent the mean ± SE (*n* = 3).

Following cellular uptake investigations, nanoparticles were loaded with an anti-infective drug. In this respect, spherical AOT-gentamicin loaded (SphG) and aspherical AOT-gentamicin loaded (AsphG) nanoparticles were formulated; InvA497 was also successfully coupled on the surface of drug-loaded aspherical (AsphIG) and spherical (SphIG) nanoparticles. The EE% and LC% of the various nanoparticle formulations is shown in Fig. 5a. The stretching procedure was seen to result in a reduction of approximately 10% with respect to the EE% of AsphIG and AsphG formulations relative to their spherical counterparts, as well as a drop in LC% of approximately 15%. The amount of InvA497 present on AsphIG and SphIG surfaces was found to be comparable (Fig. 5b), and slightly higher than that of the AsphI and SphI formulations.

The size of drug-loaded nanoparticles was also found to be similar to unloaded comparators (data not shown), however a significant decrease in the measured surface charge was found for the drug loaded nanoparticles comparing with the non-loaded formulations (Fig. S3).

**Fig. 6:**
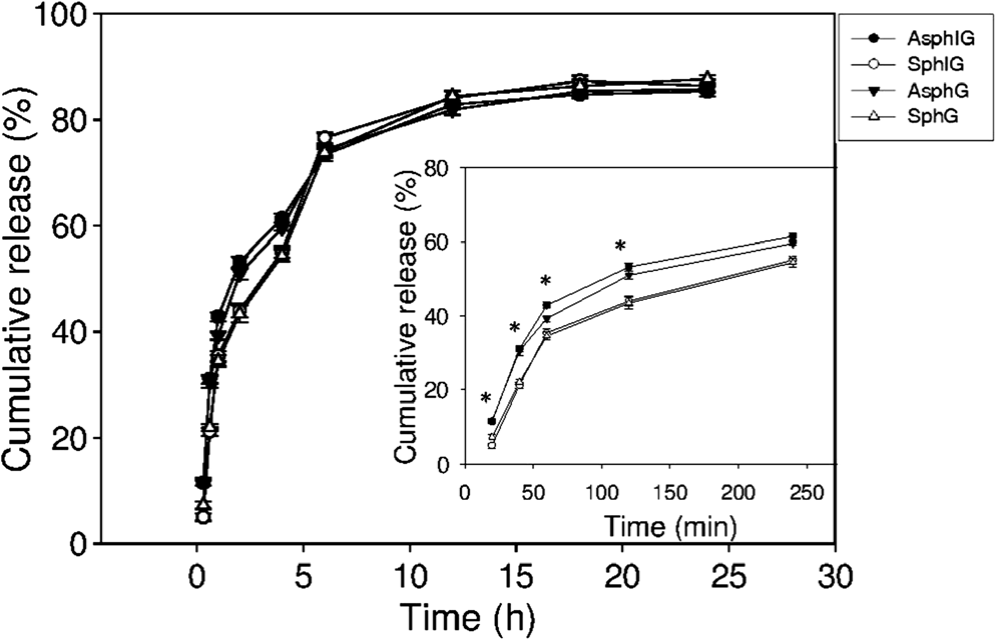
*In vitro* release kinetics. AOT-gentamicin release was measured from aspherical (AsphIG) and spherical (SphIG) functionalized nanoparticles loaded with AOT-gentamicin, and loaded non-functionalized aspherical (AsphG) and spherical (SphG) nanoparticles over 24 h in PBS at 37°C. The insert graph shows release over the first 250 min. *Indicates statistical significance with *p* value <0.05 for AsphIG or AsphG *versus* SphIG or SphG. Results represent the mean ± SE of three independent experiments, each with duplicate samples.

The *in vitro* release profiles of AOT-gentamicin from AsphIG, SphIG, AsphG and SphG were also measured at a pH of 7.4 (Fig. 6).

A slight difference in the kinetics of drug release as a function of nanoparticle shape was observed within the first two hours, with a greater release noted from the aspherical nanoparticles (AsphG and AsphIG) compared to the spherical ones; moreover an accompanying change in shape of these nanoparticles was observed to occur within this time period, resulting in a recovery of spherical character (Fig. S4). No difference was however noted in the extent of release from functionalized and non-functionalized carrier systems.

### Anti-Infective Efficacy Study

**Fig. 7:**
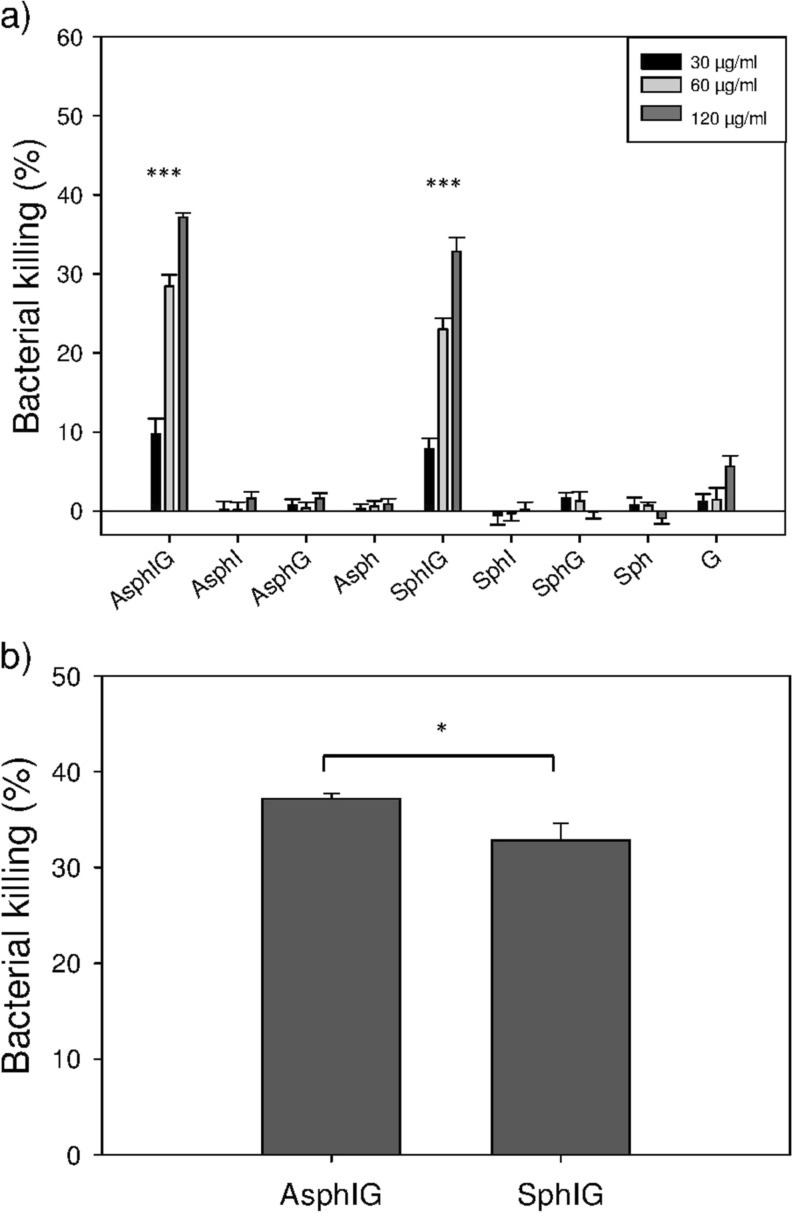
Efficacy study of nanoparticles against intracellular *S. flexneri*. (**a**) Percentage of killing of intracellular *S. flexneri* after 2 h treatment with aspherical InvA497-functionalized nanoparticles loaded with AOT-gentamicin (AsphIG), aspherical InvA497-functionalized nanoparticles (AsphI), aspherical nanoparticles loaded with AOT-gentamicin (AsphG), aspherical nanoparticles (Asph), spherical InvA497-functionalized nanoparticles loaded with AOT-gentamicin (SphIG), spherical InvA497-functionalized nanoparticles (SphI), spherical nanoparticles loaded with AOT-gentamicin (SphG), spherical nanoparticles (Sph) and AOT-gentamicin alone (G), using three different drug doses: 30 μg/ml, 60 μg/ml and 120 μg/ml. *** indicates statistical significance with a *p* value <0.001 for AsphIG *versus* AsphI, AsphG, Asph and G, and SphIG *versus* SphI, SphG, Sph and G. (**b**) Direct comparison of bacterial killing of AsphIG and SphIG, at 120 μg/ml of AOT-gentamicin. * indicates p value <0.05. Data shows the mean ± SE of 3 independent experiments, each employing duplicate samples.

HEp-2 cells were first infected with *S. flexneri* using different conditions (Fig. S5), in order to optimize the procedure for producing a model of intracellular infection. The invasion capacity of *S. flexneri* bacteria in different growth phases and at various MOI (multiplicity of infection - the ratio of bacterial to HEp-2 cells) was tested, in order to find parameters providing an optimal balance between invasion rate and used bacterial inoculum. HEp-2 cells were therefore infected for 2 h with varying amounts of bacteria, grown to either the exponential or stationary growth phase. Following infection with *S. flexneri*, HEp-2 cells were then treated for a further 2 h with free, unmodified (non-cell-permeable) gentamicin, in order kill any non-internalized bacteria. The application of *S. flexneri* grown to the exponential phase at an MOI of 25:1 was found to be the optimal condition for promoting uptake of a reproducible number of intracellular bacteria, while also avoiding overloading of HEp-2 cells (Fig. S5). After establishing an appropriate, non-toxic dose range of the various nanoparticle formulations (Fig. S6), the continued viability of HEp-2 cells following both infection with *S. flexneri* and treatment with various nanocarriers was then tested. All nanoparticle formulations were found to be non-toxic over the employed dose range, however a reduction in cell viability was observed after administration of free AOT-gentamicin (Fig. S7). Efficacy studies were subsequently performed (Fig. 7), where HEp-2 cells intracellularly infected with *S. flexneri* were treated with freshly prepared AOT-gentamicin-loaded and InvA497-functionalized nanoparticles (AsphIG and SphIG), surface-functionalized but drug-free aspherical and spherical nanoparticles (AsphI and SphI), AOT-gentamicin-loaded but non-functionalized nanoparticles (AsphG and SphG), and nanoparticles without any surface functionalization or drug loading (Asph and Sph). Free AOT-gentamicin was also employed as a control (Fig. 7a).

A dose dependent reduction in the number of intracellular bacteria was seen following treatment with either AsphIG or SphIG, with the bacterial killing induced by these formulations being significantly greater than all other formulations of corresponding shape, as well as the free AOT-gentamicin. A small but significant difference in bacteria killing was also found between the AsphIG and SphIG formations at the highest tested concentration, where a greater bacterial killing was registered for AsphIG compared to SphIG (Fig. 7b).

## Discussion

This study was initiated to investigate whether the previously shown ability of InvA497 to mediate uptake and efficacy of anti-infective loaded, lipid-based nanocarriers could be applied to more robust, polymer-based systems. Moreover, bacteriomimetic nanoparticles with an aspherical, rod-like shape, as exhibited by a number of invasive bacteria, were also studied in order to investigate the possible role of particle shape in system efficacy.

To achieve these objectives, aspherical nanoparticles were first prepared by applying a thermomechanical stress (29) to spherical nanoparticle precursors immobilized within a polymeric film, leading to a change of the spherical shape into an elongated rod-like morphology. The use of a low ratio of FA-PLGA:PLGA during spherical nanoparticle preparation ensured the production of nanoparticles with a covalently linked fluorescent label, as well as a large number of non-modified PLGA molecules available for subsequent InvA497 coupling (see below). As DLS-based size measurements rely on the assumption that detected particles have a spherical shape, the dimensions of aspherical nanoparticles were rather characterized by SEM image analysis (17). Stretching of 160 nm diameter Sph nanoparticles was seen to result in elongated Asph particles (Fig. 1) with an AR of approximately 2.6, a value which is comparable to the aspect ratio of *Yersinia* bacteria (37).

Aspherical and spherical nanoparticles were then surface-functionalized with InvA497, a *C-*terminal fragment of the Yersinia-derived invasion protein invasin. As observed previously (3,5,9), the uptake of spherical delivery systems functionalized with InvA497 is enhanced due to the presence of this bacterial protein on particle surfaces; it was therefore of interest to see whether the same effect was noted with an aspherical delivery system. In order to bind to the β_1_ integrin receptor on epithelial cells and mediate internalization, the *C-*terminal carboxyl group of InvA497 must remain free and project outwards from the particle surface. Therefore, amine functionalities of InvA497 were reacted with free PLGA carboxyl groups of both aspherical and spherical nanoparticles, in order to achieve protein coupling to particle surfaces without alteration of the integrin receptor-binding domain (Fig. 2a). Due to their non-reactive nature, a coupling agent, DMTMM, was first used to activate PLGA carboxyl groups, prior to addition and coupling of InvA497. AsphI and SphI formulations were found to have a similar amount of coupled InvA497 as well as percentage of surface occupancy (Fig. 2b and c), which allowed for an accurate comparison of their relative uptake.

Before carrying out uptake studies, the cytotoxicity of AsphI, SphI, Asph and Sph in a HEp-2 epithelial cell model was preliminarily assessed at values encompassing a feasible working range. Nanoparticle concentrations resulting in a comparable InvA497 concentration to that of the previously tested InvA497-functionalized liposomes (9), and corresponding to the maximum possible concentration of dose-able InvA497 were used (Fig. S2). Nanoparticle formulations were then incubated with HEp-2 cells to determine the cellular uptake. A previous study conducted with liposomal nanoparticles functionalized with the same InvA497 invasin fragment has shown an increased cellular uptake due to the presence of InvA497 on the surface of such nanocarriers (3,9); in accordance with this observation, InvA497 functionalization of both spherical and aspherical nanoparticles in the current work enhanced their uptake and internalization into epithelial cells, as confirmed by both CLSM (Fig. 3) and FACS analysis (Fig. 4). Interestingly, only slight differences in the kinetics of SphI and AsphI cellular uptake were seen, at earlier time points; no appreciable difference in the total extent of uptake was noted. This result is not entirely unexpected, given the aforementioned lack of enhancement of cellular uptake generally demonstrated by aspherical systems (18). In agreement with the previously conducted and mentioned study employing a liposomal formulation (3), InvA497-coated nanoparticles were found to be taken up via an energy-dependent mechanism (Fig. 4b).

Following the demonstration of an increase in cellular uptake of InvA497-functionalized nanoparticles, analogous nanoparticle formulations were then loaded with an anti-infective drug to enable further investigation of their properties and ultimately, their efficacy. The broad spectrum antibiotic gentamicin was employed for this purpose – however, due to the highly hydrophilic nature of gentamicin and the consequently low level of drug encapsulation within PLGA nanoparticles, a lipophilic preparation of gentamicin was used (AOT-gentamicin). Simple mixing of gentamicin and the surfactant AOT, followed by solvent evaporation, leads to formation of the lipophilized preparation, as a result of electrostatic interaction between the five amine groups of gentamicin molecules and AOT (36). After AOT-gentamicin preparation, AsphIG, SphIG, AsphG and SphG were formulated and characterized. Comparable results were found for the SphG nanoparticles and AOT-gentamicin loaded PLGA nanoparticles previously prepared by Imbuluzqueta *et al*. (36), with an EE% and LC% of 43 and 46% respectively. A slight reduction of approximately 10%–15% in EE% and LC% was observed for aspherical as compared to spherical formulations, as a result of the stretching procedure (Fig. 5); however even with this reduction, aspherical nanoparticle formulations still demonstrated a good ability to incorporate AOT-gentamicin. The amount of InvA497 functionalization for both AsphIG and SphIG was found to be slightly higher than for the AsphI and SphI, but remained comparable between AsphIG and SphIG despite the small differences in amount of encapsulated drug.

Next, the kinetics of AOT-gentamicin release from nanocarriers was tested (Fig. 6). A marginally faster release of AOT-gentamicin was noted from the aspherical nanoparticles as compared to the spherical ones, but only during the initial stages of the study. This slightly accelerated release profile may potentially have been driven by a recovery of spherical shape, as was noted in aspherical nanoparticle samples at the conclusion of the release study (Fig. S4). It is considered unlikely that this morphological change was rather the result of PLGA degradation, as this is expected to occur over a longer time frame of several days (38). While a reversion of aspherical particles to a spherical morphology has been previously reported (39), it has not, to the best of the authors’ knowledge, been observed to date to correlate with an accelerated release behavior; this could of course also influence the cellular uptake of such carriers, and as such constitutes a point of considerable further research interest.

In a final stage, the efficacy of functionalized and drug-loaded spherical and aspherical nanoparticles against intracellular *S. flexneri* was tested, in order to compare and ultimately evaluate the full potential of the bacteriomimetic delivery systems against a pathogen of clinical importance (40). Optimization of infection conditions and employed formulation dose was carried out prior to the conduction of efficacy studies themselves (Figs. S5 and S6). Interestingly, a negative effect on HEp-2 cell viability was seen in these optimization studies when infected cells were treated with 120 μg/ml of free AOT-gentamicin compared with all AOT-gentamicin loaded nanoparticles, indicating a benefit of incorporating AOT-gentamicin into nanoparticles regardless of their shape and surface functionalization (Fig. S7). In the efficacy study itself, AsphIG and SphIG induced a higher bacterial killing than all other tested formulations, with a dose dependent killing effect being observed (Fig. 7a). Notably, a small but significant improvement of bacterial killing was seen with AsphIG as compared to SphIG at the highest employed dose (Fig. 7b). This difference indicates a promising application of shape-modified InvA497-functionalized carrier systems for intracellular infection treatment; however the modest magnitude of this difference highlights the considerable remaining scope for carrier system development and probing of shape effects – namely, optimization of aspect ratio and maintenance of asphericity - in order to further enhance intracellular bacterial killing.

Taken together, these results demonstrate that the presence of InvA497 on the surface of polymer-based nanocarriers in combination with encapsulation of AOT-gentamicin is able to increase the ability of the delivery system to effectively treat infected epithelial cells. The obtained data on bacterial killing indicate that carrier shape may potentially have an influence on the efficacy of bacteriomimetic delivery systems; regardless of shape however, functionalization of polymeric systems with InvA497 presents a delivery option where the advantage of being actively transported across cell membranes in order to access intracellular bacteria is combined with an ability to release drug cargo within the intracellular environment.

## Conclusion

In summary, we have shown that InvA497 can successfully mediate the cellular internalization and efficacy of polymeric carrier systems against intracellular bacterial infections. So-called bacteriomimetic nanocarriers, surface functionalized with InvA497 and possessing either a spherical or aspherical shape, showed a better cellular uptake compared to nanocarriers without InvA497. Anti-infective loaded bacteriomimetic systems further demonstrated a markedly improved killing of intracellular *S. flexneri* in comparison to non-functionalized systems. The small but significant improvement in killing of intracellular *S. flexneri* resulting from treatment with a high dose of aspherical bacteriomimetic systems as compared to spherical is of great interest for future work. Subsequent work will therefore focus on the further optimization of robust, polymer-based bacteriomimetic systems. This will include greater exploration of the ability to enhance the impact of shape factors, as well as application of these systems for the control of intracellular pathogens and their respective diseases typical of specific routes of administration, such as the gastro-intestinal and pulmonary tract.

## Electronic supplementary material

ESM 1 (DOCX 1974 kb)

## Abbreviations

AOT-gentamicin: Gentamicin bis(2-ethylhexyl) sulfosuccinate sodium salt
AR: Aspect ratio
Asph: Aspherical nanocarriers
AsphG: Aspherical AOT-gentamicin-loaded nanocarriers
AsphI: Aspherical InvA497-functionalized nanocarriers
AsphIG: Aspherical AOT-gentamicin-loaded, InvA497-functionalized nanocarriers
BCA: Bicinchoninic acid
CFU: Colony forming units
DAPI: 4′,6-diamidino-2-phenylindole
DLS: Dynamic light scattering
DMTMM: 4-(4,6-Dimethoxy-1,3,5-triazin-2-yl)-4-methylmorpholinium chloride
EE%: encapsulation efficiency
FACS: Fluorescence-activated cell sorting
FA-PLGA: Fluoresceinamine-PLGA
FCS: Fetal calf serum
FSC: Forward scatter
InvA497: 497 amino acid length fragment of the C-terminal region of invasin
LC%: Loading capacity
MOI: Multiplicity of infection
NTA: Nanoparticle tracking analysis
PLGA: Poly (lactic-co-glycolic acid)
PVA: Polyvinyl alcohol
RPMI: Roswell Park Memorial Institute
SE: Standard error of the mean
SEM: Scanning electron microscopy
Sph: Spherical nanocarriers
SphG: Spherical AOT-gentamicin-loaded nanocarriers
SphI: Spherical InvA497-functionalized nanocarriers
SphIG: Spherical AOT-gentamicin-loaded, InvA497-functionalized nanocarriers
SSC: Side scatter
