# Supplementary material for: Aspherical and Spherical InvA497-Functionalized Nanocarriers for Intracellular Delivery of Anti-Infective Agents
Source: Pharm Res. 2018 Dec 5;36(1):22. doi: 10.1007/s11095-018-2521-3 (PMC6290668; doi:10.1007/s11095-018-2521-3)
Supplement: Supplementary file 1 — (DOCX 1974 kb) [file 11095_2018_2521_MOESM1_ESM.docx]

**Supplementary Material**

**Aspherical and Spherical InvA497-functionalized Nanocarriers for Intracellular Delivery of Anti-infective Agents**


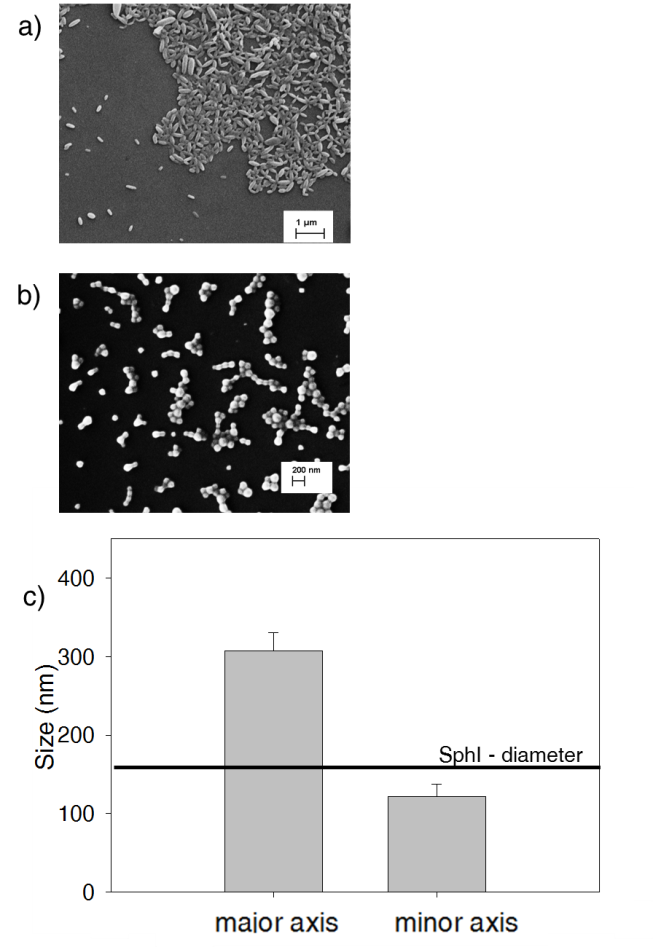


Figure S1. SEM images of aspherical (AsphI, a) and spherical (SphI, b) nanoparticles functionalized with InvA497, demonstrating the continued presence of aspherical or spherical shape after surface functionalization. The major and minor axis dimensions of aspherical nanoparticles were also assessed (c). Results represent mean ± SE (n =98).

**Script for PovRay 3.7 - Generation of 3D InvA497-functionalized nanoparticle model**

The following script was written for PovRay 3.7 to be used as an *.inc file:

//------------------ random functions standard include file------------------

#include "rand.inc" //random functions provided by PovRay

#include "invasin_tex.inc" //appearance of invasin molecules

#include "invasin_surf03.inc" //coordinates of invasin surface vertices, generated as an export file from YASARA using pdb entry 1CWV

#declare Random_1 = seed (124323); //initializing random coordinate #1

#declare Random_2 = seed (1335); //initializing random coordinate #2

#declare NP_fin_01 = finish {ambient 0 emission 0 diffuse 1 brilliance 1 subsurface { translucency <4.69, 3.69, 2.69> } specular 0.15 roughness 0.5} //appearance of particles

#declare NP_tex_03 = texture {pigment {granite colour_map {[0.0, srgb <0.6,0.6,0.61>] [1.0, srgb <0.8,0.8,0.81>]}} normal { granite 0.1 } finish {NP_fin_01} scale 0.1} texture {pigment {color srgbt<1,1,1,1>} normal { granite 0.5 } finish {ambient 0 emission 0 diffuse 0 brilliance 1 specular 0.5 roughness 0.01} scale 0.1} //layered texture of particles

#declare invasin_tex_01 = texture {pigment {rgb <0.6,0.7,0.5>} finish {phong 0.4}} //appearance of the space occupied by invasins – only seen when included as an object

//INVASINS===========================================================

#declare inv_scaled = object {invasin_surf scale 0.000001163 translate <0.0007,0,-0.033>} //model of invasin molecule, scaled to PovRay units

#declare inv_vol = cylinder{ <-0.0186/2,0,0>, <0.0186/2,0,0> ,0.00342 texture {invasin_tex_01}} //Volume which invasins occupy

//ASPHERICAL_NP====================================================

#declare NP_basic_shape01 = //--------------------------------------------------------------------------

object{ //Spheroid(CenterVector, RadiusVector Rx,Ry,Rz )

Spheroid(<0,0,0>, <0.303/2,0.1207/2,0.1207/2> )

texture{ NP_tex_03} scale<1,1,1> rotate<0, 0,0> translate<0,0.00,0>

} //shape of the aspherical naoparticle

#declare NP_surface01 = difference {object {NP_basic_shape01 scale 1.01} object {NP_basic_shape01 scale 1.005}} //Volume in which invasins are placed

#declare invasins_rand01 = merge {

#local Nr = 0; // start

#local EndNr = 198; // number of invasins on surface

#while (Nr< EndNr)

object {inv_scaled

rotate VRand_On_Sphere(Random_2)*180

translate VRand_In_Obj( NP_surface01, Random_1)*1

texture{ invasin_tex_01 } //interior_texture { PA_tex_01int } // end of texture

} // end of object

#local Nr = Nr + 1; // next Nr

#end

}

//SPHERICAL_NP=======================================================

#declare NP_basic_shape02 = sphere {<0,0,0>, 0.1912/2 texture{ NP_tex_03} scale<1,1,1> rotate<0, 0,0> translate<0,0.00,0>} //shape of the spherical naoparticle

#declare NP_surface02 = difference {object {NP_basic_shape02 scale 1.01} object {NP_basic_shape02 scale 1.005}} //Volume in which invasins are placed

#declare invasins_rand02 = merge {

#local Nr = 0; // start

#local EndNr = 235; // number of invasins on surface

#while (Nr< EndNr)

object {inv_scaled

rotate VRand_On_Sphere(Random_2)*180

translate VRand_In_Obj( NP_surface02, Random_1)*1

texture{ invasin_tex_01 } //interior_texture { PA_tex_01int } // end of texture

} // end of object

#local Nr = Nr + 1; // next Nr

#end

}

**Calculation of surface occupancy**

| gyration radius of InvA497= | | 5.5 nm | (Yasara(1)) |  |  | | |  |
| --- | --- | --- | --- | --- | --- | --- | --- | --- |
|  |  |  |  |  |  | | |  |
| aspherical | (spheroid) |  |  | spherical | (sphere) | | |  |
| r1 (b) = | 151.5 | nm |  | r1= | 95.6 | | | nm |
| r2 (a) = | 60.35 | nm |  |  |  | | |  |
| Surface formula: |  |  |  | Surface formula: | | | |  |
| 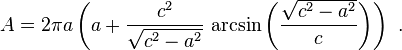   \|  \| \| --- \| |  |  |  |  | | 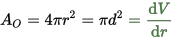 | |  |
|  |  |  |  |  | |  | |  |
| A(spheroid)= | 95 603.7676 | nm2 |  | A(sphere)= | | 114 848.585 | | nm2 |
|  |  |  |  |  | |  | |  |
| A(InvA497)= | n(InvA497) * 2πr(gyration)2 | | | A(InvA497)= | | n(InvA497) * 2πr(gyration)2 | | |
| n(InvA497)= | 198 |  |  | n(InvA497)= | | 235 |  | |
| A(InvA497)= | 37633.1384 | nm2 |  | A(InvA497)= | | 44665.5936 | nm2 | |
| A/A= | 0.39 | (= occupancy) | | A/A= | | 0.39 | (= occupancy) | |

**Cytotoxicity assessment – MTT assay**

The cytotoxicity of blank spherical and aspherical nanoparticles, with or without surface InvA497 was assessed using the (3-(4,5-di-methylthiazol-2-yl)-2,5-diphenyltetrazolium bromide) (MTT) assay (Figure S2). HEp-2 cells in 96-well culture plates were incubated with nanoparticles at total PLGA concentrations of either 0.63 or 1.06 mg/ml (corresponding to 45 or 100 µg/ml coupled InvA497 respectively, where appropriate), in RPMI 1640 medium (Gibco, Carlsbad, USA), for 4 h at 37 °C and 5% CO_2_. These two concentration levels were chosen in order to cover the determined feasible working range for later uptake and efficacy studies – the lower concentration corresponding to the concentration of InvA497 utilized previously for testing of InvA497-functionalized liposomes (2), and the higher concentration representing the most concentrated sample able to be administered in the current work. RPMI 1640 medium alone and RPMI containing 2% Triton X-100 (Sigma Aldrich, Steinheim, Germany) were employed as negative and positive controls respectively. Following 4 h of incubation the supernatant of each plate well was removed, and 10% (v/v) MTT reagent (5 mg/ml; Sigma Aldrich, Steinheim, Germany) in phosphate buffered saline (PBS) was added. After further 4 h incubation at 37 °C and 5% CO_2_, the medium was removed and formed formazan crystals were solubilized by incubation for 15 min in 100 µl of dimethyl sulfoxide (Sigma Aldrich, Steinheim, Germany). The absorbance of each plate well was then measured with a plate reader (TECAN, Männedorf, Switzerland) at 550 nm. Absorbance was then standardized to the positive control (0% of cell viability) and cell viabilities were calculated in comparison to the negative control (100% cell viability).


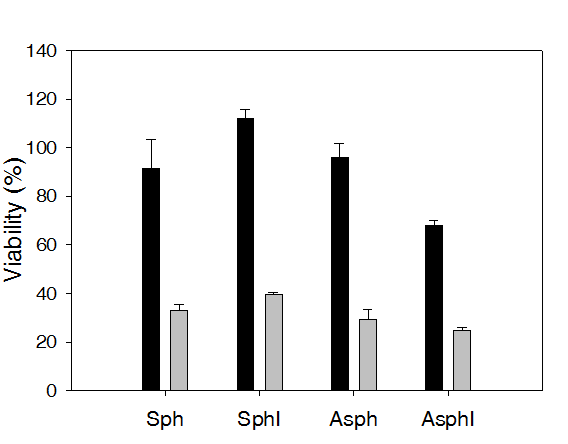


Figure S2. Viability of HEp-2 cells at the limits of the feasible working concentration range. Cells were incubated with nanoparticle formulations (spherical nanoparticles = Sph, InvA497-functionalized spherical nanoparticles = SphI, aspherical nanoparticles = Asph, and InvA497-functionalized aspherical nanoparticles = AsphI) at total PLGA concentrations of 0.63 mg/ml (black bars) and 1.06 mg/ml (grey bars), corresponding to 45 µg/ml and 100 µg/ml respectively of InvA497 in the case of functionalized systems. The employed concentrations were selected in order allow for comparison with the concentration of InvA497 previously tested in functionalized liposomes (2), as well as to investigate cell viability following exposure to the highest concentration of InvA497 able to be administered on particles without dilution. Results represent the mean±SE (n=3).


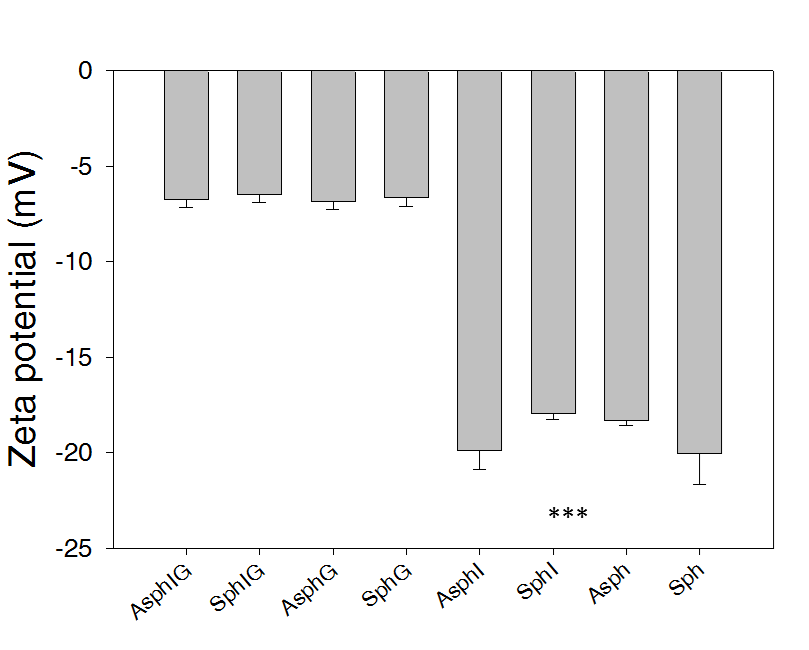


Figure S3. The zeta potential of aspherical and spherical InvA497-functionalized nanoparticles loaded with AOT-gentamicin (AsphIG and SphIG respectively), aspherical and spherical nanoparticles loaded with AOT-gentamicin (AsphG and SphG), aspherical and spherical InvA497-functionalized nanoparticles (AsphI and SphI) and aspherical and spherical nanoparticles (Asph and Sph), was measured by electrophoretic mobility. A decrease in the magnitude of surface charge was found for drug-loaded formulations.*** indicates statistical significance with a p value < 0.001 for AsphIG, SphIG, AsphG and SphG versus AsphI, SphI, Asph and Sph. Data shows the mean ± SE (n=9).


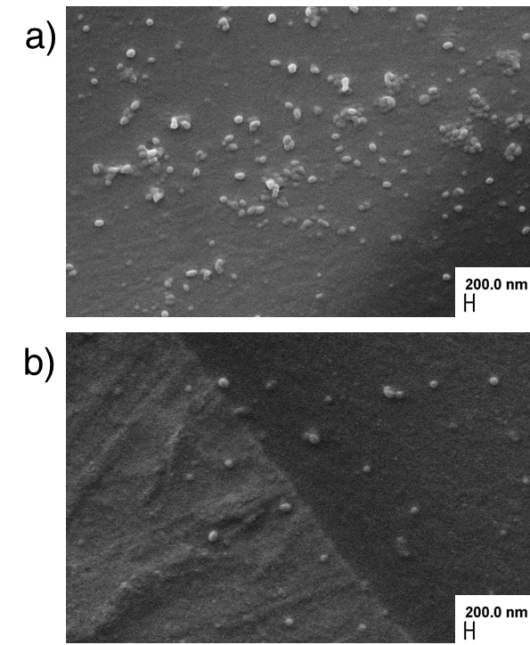


Figure S4. SEM images of aspherical nanoparticles functionalized with InvA497 and loaded with AOT-gentamicin (AsphIG, a) or without functionalization (AsphG, b) after 2 h of incubation at 37 °C during drug release studies. Changes in the aspherical shape and a recovery of the original spherical geometry were seen for both functionalized and non-functionalized aspherical nanoparticles.

**Invasion assay**

The invasion capacity of *Shigella flexneri* bacteria in different growth phases was first tested, in order to be able to employ infection conditions in later efficacy assays giving the optimal balance between invasion rate and used bacterial inoculum (Figure S5). After seeding and culturing HEp-2 cells in a 24-well plate for approximately 24 h, the cells were infected with *S. flexneri* dispersed in RPMI 1640 medium. Bacteria had previously been cultured and incubated in glass tubes containing 10 ml of Tryptic Soy Broth (TSB medium, BD, Difco, Maryland) overnight at
37 °C, with shaking to reach the stationary growth phase. Before infecting cells, *S. flexneri* cultures were freshly diluted with TSB medium and incubated at 37 °C for 2 h, to allow for growth to the exponential phase. Both overnight cultures (bacteria in stationary phase) and further 2 h cultures (bacteria in exponential phase) were used to infect HEp-2 cells. In both cases, bacterial cultures were washed once and resuspended in RPMI 1640 medium prior to addition to HEp-2 cells. Different bacterial loads, expressed as multiplicities of infection (MOI - bacteria: HEp-2 cell ratio) were used, following by centrifugation to facilitate sedimentation of bacteria onto the cells
(6 708*g* for 5 min). Culture plates were then incubated for 2 h in a humidified incubator at 37 °C and 5% CO_2_ atmosphere in order to allow for bacterial adhesion and cellular invasion. Cells were then washed with PBS and incubated for 2 h with RPMI buffer containing 50 µg/ml of gentamicin solution (Sigma-Aldrich, Steinheim, Germany) for extracellular bacteria killing. Extracellular bacteria were then removed by washing of cells with PBS. In order to count the intracellular bacteria, HEp-2 cells were lysed using 0.01% Triton X-100 (Sigma Aldrich, Steinheim, Germany) and the cell lysate was plated in sterile agar plates in serial dilutions. Plated lysates were incubated overnight at 37 °C; colony forming units (CFU) of *S. flexneri* were then counted, multiplied by relevant dilution factors and expressed as a percentage of the number of colonies from the inoculum, termed the percentage of invasion.


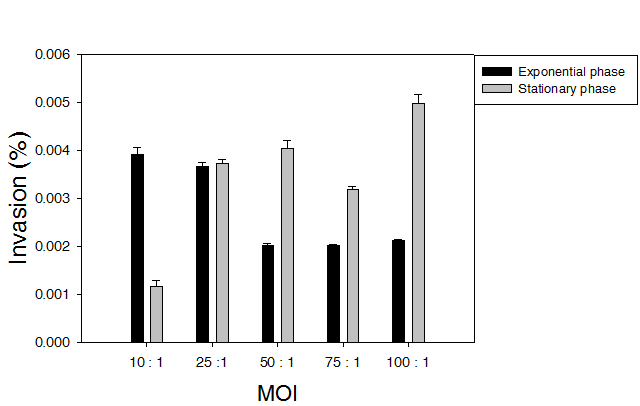


Figure S5. Percentage of invasion after 4 h incubation of *S. flexneri* with HEp-2 cells with different MOI, using bacteria in either the exponential phase or stationary phase. The percentage of invasion represents the percentage of intracellular bacterial colonies, relative to the initial *S. flexneri* bacterial colonies (CFU) used for infection. For the subsequent efficacy study, an MOI of 25:1 was selected as this condition was found to give the most consistent number of intracellular bacteria; bacteria in the exponential phase at this MOI were employed, as this resulted in the highest actual number of intracellular CFU (approximately 1.8 x 10^3^). Results represent the mean ± SE (n=6).

**Cytotoxicity assessment – lactate dehydrogenase (LDH) assay**

The viability of HEp-2 cells following treatment with various drug-loaded nanoparticle formulations was assessed using an LDH cytotoxicity detection kit (Roche, Mannheim, Germany) according to the manufacturer’s instructions (Figure S6). After seeding and culturing the HEp-2 cells in a 96-well plate, cells were incubated with different concentrations of nanoparticles (concentration of AOT-gentamicin 5-120 µg/ml, corresponding concentration of InvA497 12-50 µg/ml) dispersed in RPMI 1640 medium without phenol red (Gibco, Carlsbad, USA) for 4 h at 37 °C and 5% CO_2_. As above, RPMI 1640 medium alone and RPMI with 2% Triton X-100 served as controls. Following incubation the supernatant was removed from each plate well and the liquid was incubated with LDH reagent for
3 min at room temperature, followed by measurement of absorbance at 492 nm via a plate reader (TECAN, Männedorf, Switzerland). Absorbance was then standardized to a positive control (Triton X-100) and cell viabilities were calculated in comparison to a negative control (RPMI-treated cells).


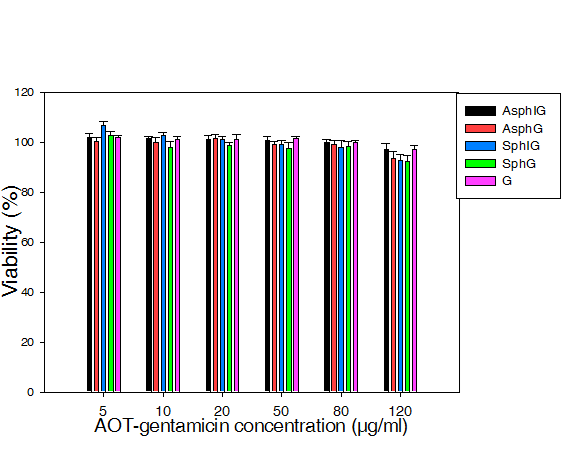


Figure S6. Viability of HEp-2 cells after 4 h treatment with aspherical InvA497-functionalized nanoparticles loaded with AOT-gentamicin (AsphIG), aspherical nanoparticles loaded with AOT-gentamicin (AsphG), spherical InvA497-functionalized nanoparticles loaded with AOT-gentamicin (SphIG), spherical nanoparticles loaded with AOT-gentamicin (SphG) and AOT-gentamicin alone (G). Cell viability over this nanoparticle concentration range was tested in order to have a content of AOT-gentamicin comparable with the previously tested gentamicin-loaded liposomes (2). Results represent the mean ± SE (n=3).

**Viability of infected cells after nanoparticle treatment**

After the infection and treatment procedure, HEp-2 cells were incubated with a 20 µg/ml solution of fluorescein diacetate (stock solution 1 mg/ml in acetone, FDA, Sigma Aldrich, Steinheim, Germany) and 40 µg/ml propidium iodide (stock solution 1 mg/ml, Sigma Aldrich, Steinheim, Germany) in PBS, in order to stain respectively live cells (green) and dead cells (red). Cells were then imaged via confocal microscopy (Figure S7). Cell viabilities were observed in comparison to a negative control (infected cells without treatment) and a positive control (infected cells treated with 2% Triton X-100, Sigma Aldrich, Steinheim, Germany).


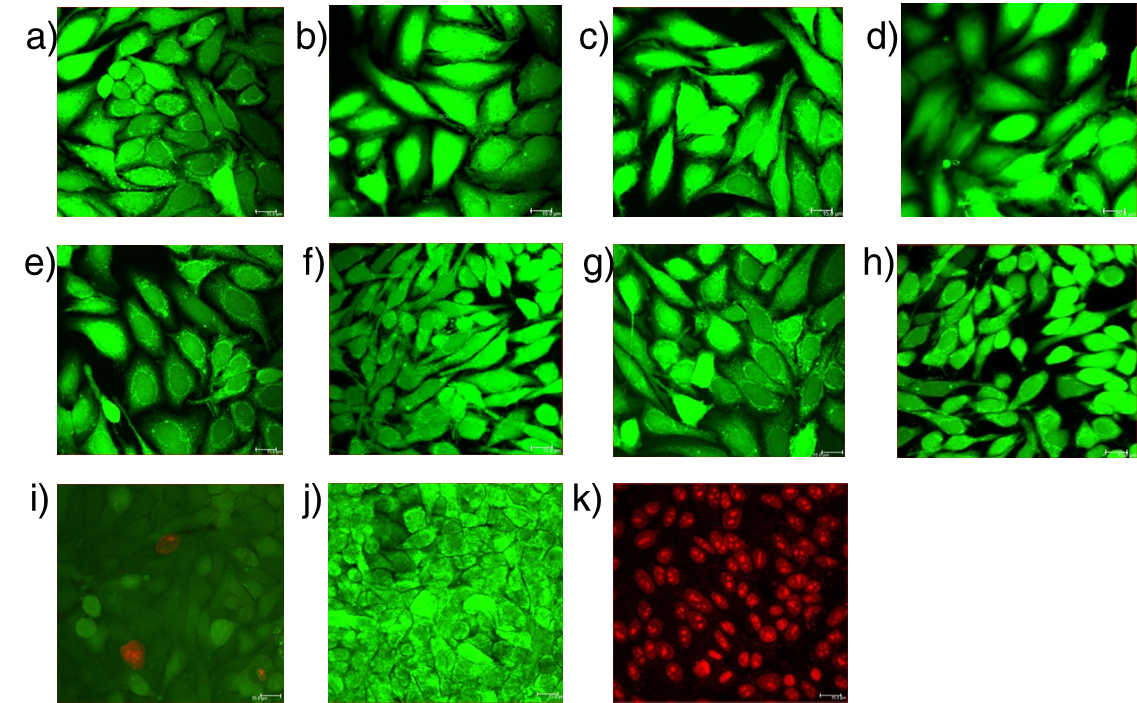


Figure S7. Viability of *S. flexneri* infected HEp-2 cells after 3 h treatment with aspherical InvA497-functionalized nanoparticles loaded with AOT-gentamicin (a), aspherical InvA497-functionalized nanoparticles (b), aspherical AOT-gentamicin loaded nanoparticles (c), aspherical nanoparticles (d), spherical InvA497-functionalized nanoparticles loaded with AOT-gentamicin (e), spherical InvA497-functionalized nanoparticles (f), spherical nanoparticles loaded with AOT-gentamicin (g), spherical nanoparticles (h) and AOT-gentamicin alone (i). Cell viabilities were observed in comparison to a negative control (infected cells without treatment, j) and a positive control (infected cells treated with 2% Triton X-100, k). Cell viability following treatment with formulations containing a total AOT-gentamicin dose of 120 µg/ml is shown. A continued viability of HEp-2 cells following both infection with *S. flexneri* and treatment with various nanoparticle formulations was seen, while a negative effect on HEp-2 cell viability was seen when infected cells were treated with 120 µg/ml of free AOT-gentamicin. Green=live cells; red=dead cells.

**References:**

1. Krieger E, Koraimann G, Vriend G. Increasing the precision of comparative models with YASARA NOVA--a self-parameterizing force field. Proteins. 2002;47(3):393-402.

2. Menina S, Labouta HI, Geyer R, Krause T, Gordon S, Dersch P, et al. Invasin-functionalized liposome nanocarriers improve the intracellular delivery of anti-infective drugs. RSC Adv. 2016;6(47):41622-9.
